# Congenital absence of lingual frenum in a non-syndromic patient: a case report

**DOI:** 10.1186/s13256-018-1966-7

**Published:** 2019-03-10

**Authors:** Raneem Felemban, Hani Mawardi

**Affiliations:** 1 King Abdul-Aziz University – Faculty of Dentistry, Jeddah, Saudi Arabia; 2 Department of Oral Diagnostic Sciences, King Abdul-Aziz University – Faculty of Dentistry, Jeddah, Saudi Arabia

**Keywords:** Congenital, Lingual frenum, Tongue

## Abstract

**Background:**

The lingual frenum is a fold of mucous membrane connecting the ventral tongue to the floor of the mouth. In general, lingual frenum serves multiple roles; its main function is to support the tongue and aid in limiting its movement in different directions. Any anatomical or functional deficiency of lingual frenum may have an impact on tongue functions based on its severity. Historically, the absence of lingual frenum was linked to multiple genetic and developmental conditions such as infantile hypertrophic pyloric stenosis, non-syndromic ankyloglossia diseases, and Ehlers–Danlos syndromes and was never reported in otherwise healthy individuals.

**Case presentation:**

We report the absence of lingual frenum in an otherwise healthy 21-year-old Middle Eastern woman diagnosed during a routine dental examination.

**Conclusion:**

To the best of our knowledge, this is the first case to be reported in the literature with similar clinical presentation. Even without a significant impact on tongue movement or speech, it is important for health practitioners to be aware of such conditions and evaluation steps for diagnosis and management.

## Introduction

Frenum is a general term frequently used to describe a fold of integument (skin) or mucous membrane that limits the movements of an organ or specific structure [1]. Several anatomical frenula are distributed throughout the human body; the lingual frenum (LF) extends from the mid ventral tongue all the way to the floor of the mouth [2, 3]. On histological examination, LF consists of a fibrodense connective tissue band wrapped with mucosa and occasionally striated muscle fibers [4]. Other anatomical structures related to LF include lingual veins, sublingual and submandibular gland papillae, as well as plica fimbriata bilaterally. LF serves multiple functions including attachment and support of ventral tongue to floor of the mouth as well as guiding tongue movement to prevent any involuntary deviation upon function [5].

Similar to other structures in the human body, anomalies of LF (for example, length or position of insertion) may have an impact on tongue function and are often linked to systemic, genetic, or developmental conditions [5]. In some cases, LF may extend from the tip of the tongue to attach to lingual gingiva in between mandibular central incisors creating ankyloglossia (tongue tie) [6]. Ankyloglossia is an anatomical deformity that causes limited tongue movement and often presents medical challenges, such as pain in breastfeeding infants with possible impact on speech if not addressed at an early age [5]. The prevalence of ankyloglossia varies depending on different diagnostic criteria and age of assessment for diagnosis and ranges between 4.2 and 10.7% [7]. Although ankyloglossia might be part of a rare syndrome (for example, X-linked cleft palate and van der Woude syndrome), it usually represents an isolated mutation in the oral cavity [8].

Other LF developmental anomalies have been reported in the literature and linked to other genetic conditions and syndromes, such as Ehlers–Danlos syndromes (EDS) and infantile hypertrophic pyloric stenosis (IHPS) [9, 10]. EDS is a genetic connective tissue disease caused by mutations in multiple genes [11]. It affects the skin mainly, in addition to bone, joints, and blood vessels [11]. IHPS is another serious congenital disease with frequent anomaly of LF [12].

We report a case of absent LF in an otherwise healthy individual with no associated medical conditions or syndromes.

## Case presentation

A 21-year-old Middle Eastern woman presented to King Abdulaziz University – Faculty of Dentistry, Jeddah, Saudi Arabia for a routine dental evaluation. Her medical history was significant for hypothyroidism secondary to thyroidectomy procedure performed 7 years ago to treat early thyroid papillary carcinoma. She received postoperative radioactive iodine as part of the treatment protocol. She had been taking thyroxin 100 mg/day since then to manage secondary hypothyroidism and had no significant allergy history. Her dental history was significant for active orthodontic treatment for the past 2 years.

**Fig. 1:**
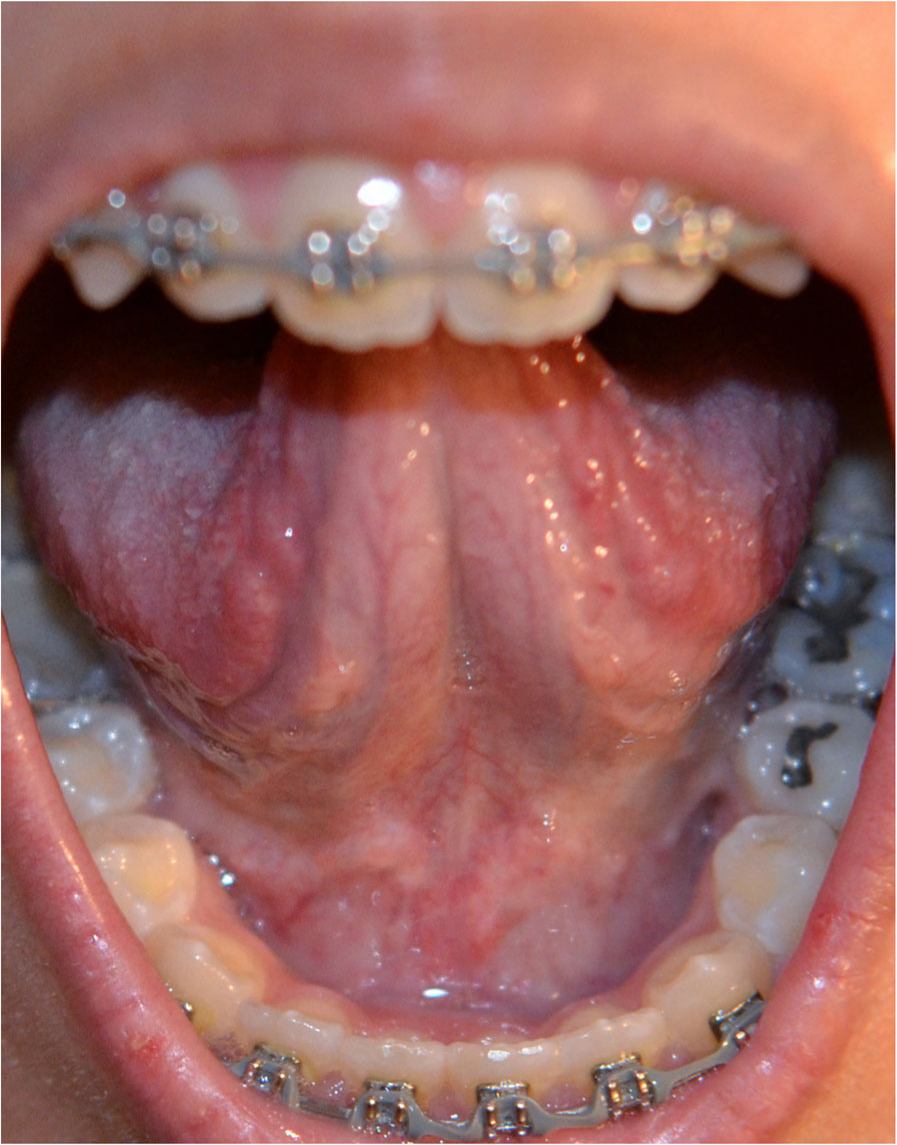
Intra-oral view demonstrating absence of lingual frenum and otherwise normal surrounding oral structures

An extra-oral examination was noncontributory with no speech impairment. An intra-oral examination was significant for complete absence of LF with normal surrounding oral structures (Fig. 1). In order to rule out a diagnosis of EDS, she was referred for medical evaluation and upon clinical examination did not meet the standard criteria for EDS. As part of the comprehensive assessment process, all family members including her six female siblings were evaluated for signs and symptoms of EDS through medical consultations and none qualified for the diagnosis. In addition, none of her family members presented with absent LF.

## Discussion

The human tongue is a muscular organ attached by multiple ligaments to the mandible, hyoid bone, styloid process, and pharynx [1]. It originates from the first, second, and third pharyngeal arches and develops at the beginning of the fourth intra-uterine week [3]. During this phase, a U-shaped sulcus develops in front of and on both sides of the oral part of the tongue which gives the tongue its mobility, except at the base of LF, which remains attached [1]. Any major disturbing event during this stage may result in a developmental defect such as ankyloglossia (that is, tongue tie) [8]. During the sixth week of gestation, and as the tongue body continues to develop, frenum-forming cells undergo apoptosis, retracting away from the tip of the tongue and giving the tongue its final mobility range [1].

In its complete developmental state, the LF is made of a band of connective tissue and covered with mucosa connecting the mid sublingual surface of the tongue to the floor of the mouth [4]. It helps in supporting the tongue and controlling its movement posteriorly; a large part of tongue functions and range of motion (for example, suction, mastication, deglutition, and speech) relies on LF shape and position [5, 13]. Often, LF may extend from the tip of the tongue to attach to lingual gingiva in between mandibular central incisors causing ankyloglossia. Complete absence of LF is another example which may result in less control of tongue movement and is linked to other syndromes such as IHPS and EDS [14–16].

IHPS is the most common condition requiring surgical intervention during the first weeks of life [17, 18]. The reported incidence of IHPS is 3 in 1000 live births, with a male to female ratio ranging from 3:1 to 6:1 [19]. The etiology of IHPS is unknown, although familial predisposition is an important feature of this condition. The exact role of surrounding environmental factors remains unclear and no available markers currently exist for identifying infants at risk for developing IHPS [20]. Clinical manifestations of IHPS typically take place 3 to 6 weeks after birth; presentation of IHPS following 3 months of age is significantly rare [21]. Typical presentation initially includes nonbilious vomiting at 4–8 weeks of age. Although vomiting may initially be infrequent, it increases over several days up to nearly every feeding [22]. De Felice *et al.* suggested using absence of LF as an early sign to diagnose IHPS due to its high prevalence [16, 23]. In this study, 25 patients with IHPS were examined for hypoplastic or absent mandibular frenum and 23 patients (92%) were found to have hypoplasia or absence of the LF [16].

EDS is a heterogeneous, multi-organ disease that can be potentially life-threatening [12]. The pathogenesis of EDS has been linked to genetic mutation which can be categorized into classical (mutation in *COL5A1* or *COL5A2* gene), hypermobility (mutation in *TNXB* gene), vascular (mutations in *COL3A1* gene), kyphoscoliosis (mutation in *PLOD1* gene), dermatosparaxis (mutations in *ADAMTS2* gene), and arthrochalasia type (mutations in *COL1A1* or *COL1A2* gene) [24]. Diagnosing EDS early in life is a major necessity to reduce any future impact on affected patients and implementation of an appropriate therapy tailored to each case. The diagnosis of EDS is mainly a clinical one using scoring of involved organs according to Beighton Hypermobility Score (Table 1) [25, 26]. However, patients often may show no significant findings other than vascular involvement (aneurysm or spontaneous arterial dissection) which is a major criterion [27].

**Table 1:** The Beighton Hypermobility Scoring system is designed to quantify joint laxity and hypermobility. It uses a simple 9-point system, where the higher the score the higher the laxity. The threshold for joint laxity in a young adult ranges from 4 to 6. Any score above 6 indicates hypermobility, but is not necessarily true [25, 26]

Joint	Finding	Points
Left little (fifth) finger	Passive dorsiflexion beyond 90°	1
	Passive dorsiflexion ≤ 90°	0
Right little (fifth) finger	Passive dorsiflexion beyond 90°	1
	Passive dorsiflexion ≤ 90°	0
Left thumb	Passive dorsiflexion to the flexor aspect of the forearm	1
	Cannot passively dorsiflex thumb to flexor aspect of the forearm	0
Right thumb	Passive dorsiflexion to the flexor aspect of the forearm	1
	Cannot passively dorsiflex thumb to flexor aspect of the forearm	0
Left elbow	Hyperextends beyond 10°	1
	Extends ≤ 10°	0
Right elbow	Hyperextends beyond 10°	1
	Extends ≤ 10°	0
Left knee	Hyperextends beyond 10°	1
	Extends ≤ 10°	0
Right knee	Hyperextends beyond 10°	1
	Extends ≤ 10°	0
Forward flexion of trunk with knees fully extended	Palms and hands can rest flat on the floor	1
	Palms and hands cannot rest flat on the floor	0

EDS affects the skin, bone, joints, and blood vessels as well as complete absence of LF [11]. EDS clinical alterations have been observed in approximately 90% of patients who are below 40 with average survival rate of 40–50 years of age [12]. A list of EDS common clinical features can be found in Table 2 [10, 12]. Common oral findings include gingival recession and Gorlin sign (tongue hypermobility) [11]. The absence of lingual and/or labial frenum is a common clinical feature, which was reported in the literature as a unique diagnostic criterion. De Felice *et al.* reported a series of 12 patients with EDS compared to 154 non-syndromic patients (that is, no known congenital malformations, chromosomal abnormalities, and they had no history of either inherited connective tissue disorders or IHPS) [14]. As none of the control group demonstrated oral anomaly or any features for EDS, it was concluded that the absence of labial frenum and/or LF is associated with EDS (100% sensitivity and 99.4% specificity). As for the absence of LF alone, it corresponded to 71.4% of sensitivity and 100% of specificity. Machet *et al.* conducted a case-control study of patients with EDS (*N* = 43) matched with controls (*N* = 86) and included evaluation of their oral frenum [28]. Out of 43 patients, 4 patients had classical EDS, 19 with hypermobile EDS, and 20 with vascular-type EDS. It was concluded that the sensitivity of absence of mandibular labial frenum was 42% and 53.5% for LF.

**Table 2:** Common clinical features associated with Ehlers–Danlos syndromes [10, 12]

Systemic criteria	• Skin involvement ◦ Hyperextensibility of skin ◦ Translucent skin ◦ Atrophic “cigarette paper” ◦ Skin scarring ◦ Subcutaneous nodules • Joint hypermobility • Cicatrization disorders • Easy bruising • Eyelid extensibility (Metenier’s sign) • Pseudotumors • Postural acrocyanosis
Temporomandibular joint and intra-oral criteria	• Recurrent subluxation of the temporomandibular joint • Tongue hypermobility (Gorlin’s sign) • Absence of lingual frenulum

Clinical examination, including the oral cavity, is key in patients’ workup to reach the proper diagnosis and eventual management when suspecting IHPS or EDS. In the current case, detailed history and examination were obtained in order to rule out EDS and IHPS in the absence of LF. All clinical features for EDS or features suspicious for other syndromes were absent other than the absent LF. Considering that the diagnosis of EDS is based on clinical findings, the current case was diagnosed as absence of LF in an otherwise healthy non-syndromic individual. To the best of our knowledge, this is the first case to be reported in the literature with similar clinical presentation. Even without a significant impact on tongue movement or speech, it is important for health practitioners to be aware of such conditions and evaluation steps for diagnosis and management.

## Conclusion

Absence of LF is commonly associated with EDS and other congenital syndromes. However, this clinical finding could also be reported in otherwise healthy patients. We report a case of a patient with absent LF and no signs of EDS or other congenital or developmental diseases.
